# 2-aminoimidazoles collapse mycobacterial proton motive force and block the electron transport chain

**DOI:** 10.1038/s41598-018-38064-7

**Published:** 2019-02-06

**Authors:** Albert Byungyun Jeon, David F. Ackart, Wei Li, Mary Jackson, Roberta J. Melander, Christian Melander, Robert B. Abramovitch, Adam J. Chicco, Randall J. Basaraba, Andrés Obregón-Henao

**Affiliations:** 1 Mycobacteria Research Laboratories, Department of Microbiology, Immunology, and Pathology, Colorado State University, Fort Collins, Colorado, 80523 USA; 2 Department of Chemistry, North Carolina State University, Raleigh, North Carolina 27695 USA; 3 Department of Microbiology and Molecular Genetics, Michigan State University, East Lansing, Michigan 48824 USA; 4 Department of Biomedical Sciences, Colorado State University, Fort Collins, Colorado, 80523 USA; 5 Present Address: College of Veterinary Medicine, University of Florida, 2015 SW 16th Ave, Gainesville, Florida 32608 USA; 6 Present Address: Department of Chemistry & Biochemistry, University of Notre Dame, 251 Nieuwland Science Hall, Notre Dame, Indiana 46556 USA

## Abstract

There is an urgent need to develop new drugs against tuberculosis. In particular, it is critical to target drug tolerant *Mycobacterium tuberculosis* (*M*. *tuberculosis*), responsible, in part, for the lengthy antibiotic regimen required for treatment. We previously postulated that the presence of *in vivo* biofilm-like communities of *M*. *tuberculosis* could contribute to this drug tolerance. Consistent with this hypothesis, certain 2-aminoimidazole (2-AIs) molecules with anti-biofilm activity were shown to revert mycobacterial drug tolerance in an *in vitro M*. *tuberculosis* biofilm model. While exploring their mechanism of action, it was serendipitously observed that these 2-AI molecules also potentiated β-lactam antibiotics by affecting mycobacterial protein secretion and lipid export. As these two bacterial processes are energy-dependent, herein it was evaluated if 2-AI compounds affect mycobacterial bioenergetics. At low concentrations, 2B8, the lead 2-AI compound, collapsed both components of the proton motive force, similar to other cationic amphiphiles. Interestingly, however, the minimum inhibitory concentration of 2B8 against *M*. *tuberculosis* correlated with a higher drug concentration determined to interfere with the mycobacterial electron transport chain. Collectively, this study elucidates the mechanism of action of 2-AIs against *M*. *tuberculosis*, providing a tool to better understand mycobacterial bioenergetics and develop compounds with improved anti-mycobacterial activity.

## Introduction

Numerous compounds targeting bacterial DNA replication, RNA transcription or transduction, and protein or cell wall biosynthesis, were developed during the explosion of antibiotic discovery between 1950s and 1980s^1^. One caveat to the effectiveness of these antibiotics is the fact they predominantly target actively growing bacteria in which these anabolic processes are at peak activity. Unfortunately, it has become evident that several clinically important infectious diseases are caused by pathogens that may persist in a lower metabolic state, thus refractory to such antibiotics. Tuberculosis (TB), in particular, is notorious for a protracted treatment regimen, requiring six months to two years with a combination of multiple antibiotics in order to eradicate susceptible and drug resistant *Mycobacterium* (*M*.) *tuberculosis*^2^, respectively. The reason(s) why TB treatment is unique amongst bacterial infections is probably multifactorial, including: the impermeable nature of the mycobacterial cell envelope^3–5^; the existence of non-replicating ‘persisters’^6^; the presence of heterogeneous bacterial populations residing in different microenvironments^7^; the stochastic activation of mycobacterial genes required to activate pro-drugs^8^; and the presence of extracellular mycobacteria forming biofilm-like communities in necrotic granulomas^9^, amongst others. Developing alternative antimicrobials or adjuncts that shorten TB therapy by targeting drug tolerant and drug resistant *M*. *tuberculosis* is, therefore, warranted.

It has recently been determined that pathogens’ cell membranes and bioenergetics represent effective therapeutic targets^10^. Its value stems from the essential role played by the electron transport chain (ETC) to generate and maintain proton motive force (PMF), required for numerous biological processes. In terms of antibiotic discovery, mycobacterial bioenergetics is a highly promising yet, until recently, unexplored field. In the seminal studies describing the discovery of clofazimine (CFZ), it was initially suggested that its mechanism of action was dependent on mycobacterial respiration^11^. However, this line of research was not pursued until recently, when two key observations ultimately provided a better understanding of therapeutic targeting *M*. *tuberculosis* energy metabolism: (a) mycobacterial ATP synthase can be specifically targeted with bedaquiline (BDQ)^12^, and (b) mycobacterial persistence depends on maintaining respiration, PMF and ATP synthesis^13–16^. Since then, several compounds have been developed to target unique aspects of mycobacterial bioenergetics and ETC, and are currently in different stages of development or clinical trials. For instance, inhibition of menaquinone biosynthesis with Ro 48-8071^17^, is an attractive target since menaquinone represents the main, perhaps only, electron carrier in the *M*. *tuberculosis* ETC. Moreover, NADH oxidation in mycobacteria is predominantly catalyzed by type-2 complex I, NADH dehydrogenase 2 (NDH-2)^18^, which is not present in humans^19^. Indeed, this complex was recently shown to be targeted by CFZ^20^ and thioridazine (TRZ)^21^, explaining the long-held observation that *M*. *tuberculosis* is inhibited by phenothiazines^22^. Lastly, dramatic bacterial clearance was observed in a murine model of TB when both terminal oxidases were simultaneously inhibited via mutagenesis (to inactivate cytochrome *bd* oxidase), and pharmacologically with Q203 (targeting cytochrome *bc*_*1*_:*aa*_*3*_ supercomplex)^23^. Taken together, these results validate mycobacterial bioenergetics as a therapeutic target.

Bacilli remaining after antibiotic therapy of *M*. *tuberculosis*-infected guinea pigs have been identified in pulmonary lesions, specifically within necrotic granulomas^9^. As these bacilli formed extracellular clusters closely apposed to host DNA, it was proposed that a driver of antibiotic tolerance in TB is the *in vivo* existence of mycobacterial biofilm-like communities^24^. It is well documented that bacteria residing in biofilms are extremely tolerant to antibiotics that are otherwise highly effective against planktonic cells^25^. The development of anti-biofilm compounds has consequently gained considerable interest in recent years^26,27^, yet biofilm formation by *M*. *tuberculosis* remains controversial. Consistent with this strategy, 2-aminoimidazole (2-AI) compounds with known anti-biofilm activity were tested and a subset were proven to effectively revert antibiotic tolerance in an *in vitro*, *M*. *tuberculosis* biofilm model^28^. While further characterizing the mechanism of action of 2-AI compounds against *M*. *tuberculosis*, it was serendipitously observed that some 2-AI compounds, in addition, potentiated the activity of β-lactam antibiotics by compromising protein secretion and lipid export^29^. As these two processes are energy-dependent^30,31^, it was posited that these 2-AI compounds could be targeting mycobacterial bioenergetics, similar to other drugs also having a cationic amphiphilic structure^32^. Using whole cell assays and inverted membrane vesicles (IMVs), herein it is shown that certain 2-AI compounds collapse components of the mycobacterial PMF and interfere with electron transport.

## Results

### 2B8 collapses both components of the mycobacterial PMF

**Figure 1:**
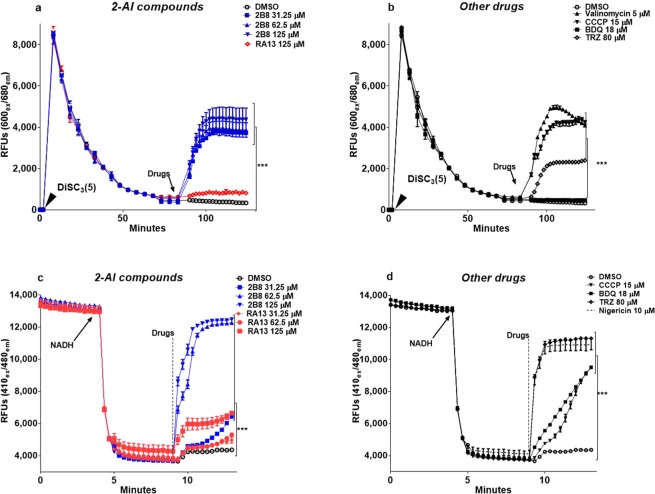
2B8 collapses both components of the mycobacterial PMF: Δψ and ΔpH. The fluorescent dyes DiSC_3_(5) and ACMA were used to evaluate 2B8’s effect on mycobacterial Δψ (**a**,**b**) and ΔpH (**c**,**d**), respectively. Uptake by live *M*. *smegmatis* slowly quenched DiSC_3_(5) fluorescence (**a**,**b**). Likewise, ACMA fluorescence was quenched upon energizing *M*. *smegmatis* IMVs with NADH (arrow), to create a ΔpH (**c**,**d**). Thereafter, bacteria or IMVs were treated with different drugs and monitored for fluorescence reversal. As indicated by fluorescence reversal, treatment with 31.25–125 µM 2B8 abruptly depolarized *M*. *smegmatis* membrane potential (**a**) and collapsed the ΔpH generated by *M*. *smegmatis* IMVs (**c**). Both parameters were also affected by 15 µM CCCP and 80 µM TRZ (**b**,**d**). The ΔpH (**d**) but not Δψ (**b**), was collapsed with 18 µM BDQ. A similar pattern was observed for 125 µM RA13 (**a**,**c**). 5 µM valinomycin and 10 µM nigericin were used as positive controls for Δψ and ΔpH. Experiments were repeated three separate times and representative data are shown. ***p < 0.001 by ANOVA.

The MIC values against *M*. *tuberculosis* are provided in Table 1 and the structures for 2B8 and RA13 in Supplementary Fig. S1. Based on our previous findings that 2B8 inhibits *M*. *tuberculosis* cell wall lipid export and protein secretion^29^, it was hypothesized that 2B8 could collapse the PMF required for these cellular processes^30,31^. PMF is collectively established by two parameters: Δψ and ΔpH^33^, with Δψ having a preponderant role in mycobacteria^13,34^. Thus, the effect of 2-AI compounds on *M*. *smegmatis* PMF was evaluated using the membrane potential and pH gradient-sensitive dyes DiSC_3_ (5) and ACMA, respectively. Upon treatment, the three tested concentrations of 2B8 (31.25–125 µM), rapidly depolarized the membrane potential of live *M*. *smegmatis* (Fig. 1a) and collapsed the ΔpH generated by *M*. *smegmatis* IMVs energized with NADH (Fig. 1c). A similar result was observed with TRZ and CCCP (Fig. 1b,d). In contrast, 125 µM RA13 had no effect on the Δψ (Fig. 1a), and its effect on ΔpH was similar to that induced by 31.25 µM 2B8 (Fig. 1c). Finally, 18 µM BDQ collapsed ΔpH (Fig. 1d), but preserved Δψ (Fig. 1b), as previously reported^35,36^. Altogether, these results suggested that 2B8 rapidly collapses components of the mycobacterial PMF, whereas RA13 is significantly less potent.

**Table 1:** MIC of compounds targeting mycobacterial bioenergetics.

compound	MIC^a^
Carbenicillin^b^	1000 μg/ml
Meropenem^b^	8 μg/ml
2B8^b^	250 µM
RA13	1000 μM
CCCP	20 µM
BDQ	0.125 µM
TRZ	62.5 µM

^a^MIC_95_ as determined in^29^.

^b^MIC of Carbenicillin, Meropenem and 2B8 against *M*. *tuberculosis* were previously reported in^29^.

Experiments were repeated three times and performed in triplicate.

### 2B8 increases mycobacterial oxygen consumption

In normoxic conditions, oxygen, the final electron acceptor, is reduced by electrons flowing through the ETC to generate water. The energy released by electron flow is harnessed to pump protons outside the cell membrane and form a proton gradient (or PMF) that drives the enzymatic phosphorylation of ADP by ATP synthase^37^. In this process, known as oxidative phosphorylation, oxygen consumption is coupled to ATP synthesis^37^. Conversely, uncoupling occurs when increased oxygen consumption is not conducive to ATP synthesis. The prototypical uncoupler CCCP collapses the PMF by shuttling protons back from outside the cell membrane, to the inside^38^. In an effort to reestablish the PMF, the ETC is driven into a futile cycle in which oxygen consumption is increased but ATP synthesis is not commensurate.

**Figure 2:**
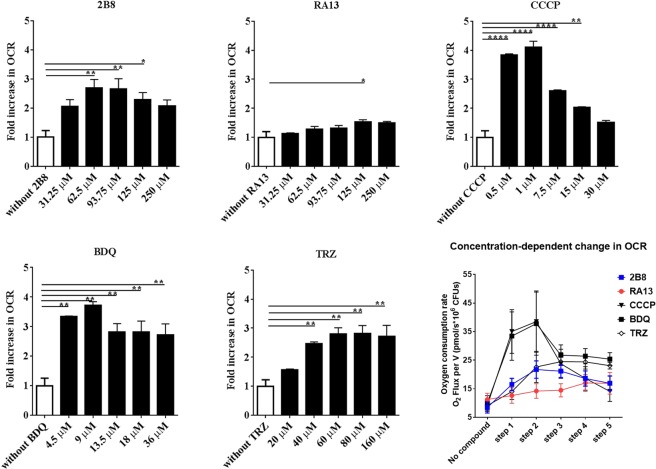
2B8 increases *M*. *tuberculosis* respiration at low concentrations. Oxygen consumption of *M*. *tuberculosis* mc^2^ 6206 strain was monitored in real-time using high-resolution respirometry. Upon bacterial inoculation, the oxygen consumption rate (OCR) was measured at steady state (white columns, without compounds). Compounds were added in stepwise increments (depicted under each drug) and the change in OCR reported as fold-increase compared to the steady state without treatment. The lower right panel is a composite of the actual OCR levels achieved after stepwise increments in compound concentration, as depicted for respective compounds (for clarity, statistical significance and compound concentrations were not included for the lower right panel). Treatment with 2B8 and CCCP resulted in a bell-shaped effect: increased OCR was observed at lower concentrations, whereas higher concentrations resulted in OCR levels similar to no treatment. RA13 induced minimal changes in OCR, with only a small increase at 125 µM. BDQ increased OCR until 9 µM and this was maintained (albeit with a decreasing trend), upon treatment with additional BDQ. The OCR increase induced by TRZ was sustained at all the tested concentrations. Experiments were repeated three different times and all biological replicates were analyzed together. *p < 0.05, **p < 0.01, ***p < 0.001 by ANOVA.

Knowing 2B8 collapsed components of the PMF, it was predicted 2B8 would increase mycobacterial oxygen consumption. Indeed, *M*. *tuberculosis* OCR increased in the presence of 2B8 (Fig. 2), leveling off at 62.5 µM 2B8. Thereafter, increasing concentrations of 2B8 resulted in decreasing *M*. *tuberculosis* OCR, such that no statistical difference was seen between basal OCR (without 2B8) and that induced by 250 µM 2B8 (highest tested concentration). A similar bell-shaped, dose-response effect was also observed for CCCP (Fig. 2). However, CCCP’s effect was more potent than 2B8 as evidenced by the greater magnitude in fold-change and absolute OCR levels, as well as the lower compound concentration required to increase *M*. *tuberculosis* OCR. Meanwhile, BDQ (as previously shown in^35,36^) and TRZ also significantly increased *M*. *tuberculosis* OCR, and this change persisted in the face of increasing compound concentration (Fig. 2). The maximum, absolute OCR levels induced by BDQ and TRZ were comparable to those induced by CCCP and 2B8, respectively (Fig. 2, bottom right panel). Finally, and consistent with the feeble effects on mycobacterial PMF, RA13 induced minimal changes in OCR (Fig. 2). These experiments were primarily performed in media without BSA, as its presence shifted the dose-response curve to the right and increased compounds’ MICs (Supplementary Table S1), possibly due to drug-protein interactions.

### 2B8 depletes intracellular ATP levels in *M. tuberculosis*

**Figure 3:**
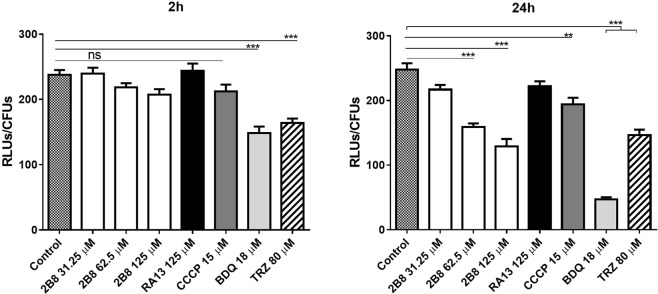
2B8 reduces intracellular ATP levels in *M*. *tuberculosis*. Intracellular ATP levels in *M*. *tuberculosis* were quantified by RLUs at 2 and 24 h after treatment. Number of CFUs was also determined in parallel and data normalized to RLUs/CFUs. After 2 h treatment, only BDQ and TRZ significantly reduced *M*. *tuberculosis* intracellular ATP levels. After 24 h, in addition to BDQ and TRZ, intracellular ATP was also reduced with 62.5 and 125 µM 2B8, and CCCP. In contrast, RA13 had no effect. Experiments were repeated three separate times and all replicates were pooled together for analysis. **p < 0.01, ***p < 0.001 by ANOVA.

To confirm that 2B8 has uncoupling activity (PMF collapse, increase OCR, ATP deficit), the amount of intracellular ATP was determined by luminescence in treated *M*. *tuberculosis* (luminescence was normalized to bacterial counts remaining after treatment). After 2 h treatment, 2B8 did not significantly affect intracellular ATP levels in *M*. *tuberculosis* (Fig. 3). Similarly, ATP levels were also unchanged in bacteria treated for 2 h with CCCP, despite rapidly collapsing both components of the PMF (Fig. 1), and potently increasing OCR (Fig. 2). At 24 h of treatment, however, ATP levels were lower in *M*. *tuberculosis* treated with CCCP or increasing concentrations of 2B8 (Fig. 3). As expected based on its mechanism of action (mycobacterial ATP-synthase inhibitor^12^), 18 µM BDQ significantly reduced ATP levels at both time points (Fig. 3). A similar effect was also observed for 80 µM TRZ (Fig. 3). In contrast, ATP levels were not affected at either time point when *M*. *tuberculosis* was treated with RA13 (Fig. 3). Altogether, the effects on components of the PMF, OCR and ATP confirmed that 2B8 has uncoupling activity in mycobacteria.

### 2B8 increases the NADH/NAD^+^ ratio in *M. tuberculosis*

**Figure 4:**
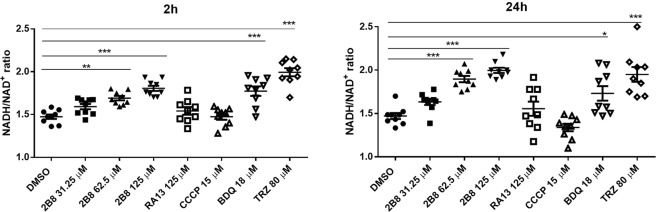
2B8 increases *M*. *tuberculosis* NADH/NAD^+^ ratio. After 2 or 24 h treatment, intracellular NADH and NAD^+^ concentrations were determined and the NADH/NAD^+^ ratio was calculated. Treatment with 62.5 and 125 µM 2B8 for 2 h resulted in a significant increase of the NADH/NAD^+^ ratio. Treatment with BDQ and TRZ also increased the ratio significantly. In contrast, 125 µM RA13 did not. After 24 h treatment, 62.5 and 125 µM 2B8, BDQ, and TRZ resulted in a significantly elevated NADH/NAD^+^ ratio. However, RA13 and CCCP were ineffective at changing the NADH/NAD^+^ ratio. Experiments were repeated three separate times and all replicates were pooled for analysis. *p < 0.05, **p < 0.01, ***p < 0.001 by ANOVA.

To determine if 2B8 affected mycobacterial redox homeostasis, the NADH/NAD^+^ ratio was evaluated. Indeed, the intracellular NADH/NAD^+^ ratio was significantly increased as early as 2 h after treating *M*. *tuberculosis* with 62.5–125 µM 2B8 (Fig. 4) and this effect persisted at 24 h (Fig. 4). This ratio was also increased at both time points when *M*. *tuberculosis* was treated with TRZ (Fig. 4), an expected finding taking in consideration that TRZ and other phenothiazines inhibit NADH oxidation by NDH-2^21^, the dominant mycobacterial complex I^18^. Interestingly, treatment with CCCP did not increase the NADH/NAD^+^ ratio at either time point (Fig. 4), despite having similar uncoupling effects as 2B8 (Figs 1–3). Again, 125 µM RA13 was inactive. Consistent with previous results^36,39^, BDQ increased the NADH/NAD^+^ ratio at both time points (Fig. 4). Altogether, these results suggested that when tested at higher concentrations, the mechanism of action of 2B8 is not limited to an uncoupling effect. The acute effects of 2-AI compounds on mycobacterial redox homeostasis were confirmed by observing a concentration-dependent decrease in the rate of alamarBlue^®^ reduction (Supplementary Fig. S2)^40^.

### Inhibition of NADH oxidation by 2B8 is restored by CFZ

**Figure 5:**
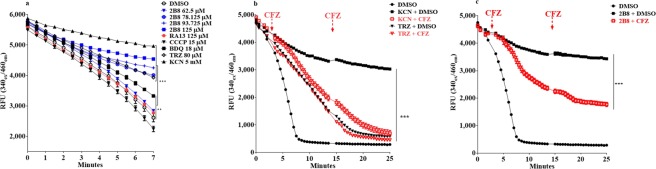
Inhibition of NADH oxidation by 2B8 is partially restored by CFZ. (**a**) NADH oxidation by *M*. *smegmatis* IMVs was evaluated by monitoring the rate of fluorescence decay at 340_ex_/460_em_. Treatment with 2B8 at 78.125–125 µM, 5 mM KCN and 80 µM TRZ potently inhibited NADH oxidation. In contrast, NADH oxidation was minimally inhibited by a lower concentration of 2B8 (62.5 µM) or 125 µM RA13. BDQ at 18 µM had an intermediate inhibitory activity, whereas NADH oxidation was accelerated with 15 µM CCCP. (**b**,**c**). Restoration of NADH oxidation by clofazimine (CFZ) was measured in *M*. *smegmatis* IMVs treated with 5 mM KCN (**b**), 80 µM TRZ (**b**), or 125 µM 2B8 (**c**). Addition of 42 µM CFZ (dotted lines) restored NADH oxidation in KCN-treated IMVs (**b**). As expected, CFZ did not restore NADH oxidation in TRZ-treated IMVs (**b**). In contrast, CFZ also restored NADH oxidation in 2B8-treated IMVs (**c**), albeit partially in comparison to KCN-treated samples. Experiments were done three separate times and representative data are shown. ***p < 0.001 by ANOVA.

To directly test whether 2B8 increased the NADH/NAD^+^ ratio by inhibiting NADH oxidation, NADH decay catalyzed by *M*. *smegmatis* IMVs was evaluated by fluorescence at 340_ex_/460_em_^41^. NADH oxidation was almost completely inhibited by 5 mM KCN (Fig. 5a,b), confirming that NADH oxidation by *M*. *smegmatis* IMVs was performed by enzymes of the ETC and not cytoplasmic enzymes contaminating the IMVs. As expected, an inhibitor of NDH-2 such as TRZ^21^, significantly slowed down NADH oxidation (Fig. 5a,b). In contrast to KCN, however, NADH oxidation in the presence of TRZ progressed almost to completion when followed for an extended period of time (Fig. 5b), possibly catalyzed by mycobacterial type-1 NADH dehydrogenase^18^. Consistent with the effects on the NADH/NAD^+^ ratio in intact mycobacteria (Fig. 4), NADH oxidation by *M*. *smegmatis* IMVs was significantly inhibited at concentrations greater than 62.5 µM 2B8 (Fig. 5a). Inhibition of NADH oxidation by 125 µM 2B8 was similar to KCN in that it persisted over an extended period of time (Fig. 5c). As previously reported^36,39^, NADH oxidation by mycobacterial IMVs was also inhibited by 18 µM BDQ (Fig. 5a), consistent with the increased NADH/NAD^+^ ratio induced by BDQ in live *M*. *tuberculosis* (Fig. 4). Finally, it was observed that 125 µM RA13 did not have any effect, while 15 µM CCCP even had the tendency to accelerate NADH oxidation (Fig. 5a), however this was not statistically significant. These results suggested 2B8 could be increasing the NADH/NAD^+^ ratio indirectly by inhibiting the ETC downstream, similar to KCN. Alternatively, 2B8 treatment could be blocking NADH oxidation by inhibiting mycobacterial NADH dehydrogenases^18^.

To explore these possibilities, it was evaluated whether CFZ could restore NADH oxidation when inhibited by 2B8. It was previously shown that in the presence of a reduced quinone pool (i.e. KCN treatment), CFZ restores NADH oxidation by serving as a surrogate electron acceptor in an NDH-2 catalyzed reaction^20^. However, this would not occur in the presence of an NDH-2 inhibitor such as TRZ^20^. As reported^20^, NADH oxidation by IMVs exposed to KCN was restored upon addition of CFZ, with NADH oxidation essentially reaching completion (Fig. 5b). In contrast, the rate of NADH oxidation in the presence of TRZ was not altered by CFZ, with both curves superimposing (Fig. 5b). NADH oxidation in the presence of 2B8 was also restored by CFZ (Fig. 5c), albeit to a lower extent than observed for KCN (Fig. 5b), as evidenced by the fact that oxidation did not progress to completion during the assayed time (Fig. 5c). This result with CFZ indicated that 2B8’s effect on NADH oxidation is not through direct inhibition of mycobacterial NDH-2.

### 2B8 inhibits the ETC in *M. smegmatis* IMVs

**Figure 6:**
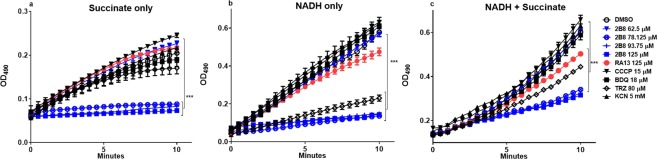
2B8 inhibits the ETC of mycobacterial IMVs. The ETC of mycobacterial IMVs was initiated with 130 mM succinate (**a**), 1 mM NADH (**b**) or NADH + succinate (**c**), and was evaluated by measuring INT reduction at 490 nm. (**a**) Out of the tested compounds, only 2B8 (78.12–125 µM), was able to inhibit INT reduction when the ETC was energized with succinate. (**b**) As expected, 80 µM TRZ inhibited INT reduction when the ETC was energized with NADH. INT reduction in the presence of NADH was also inhibited by 2B8. RA13 had a lower inhibitory effect. (**c**) When the ETC was activated with both NADH and succinate, 2B8 had the most potent inhibitory effect, followed by TRZ and RA13. The other tested compounds did not affect INT reduction. Experiments were repeated three separate times and representative data are shown. *p < 0.05, **p < 0.005, ***p < 0.001 by ANOVA.

To specifically evaluate 2B8’s effects on the mycobacterial ETC, *M*. *smegmatis* IMVs were energized with NADH, succinate or both, and INT reduction was monitored over time. Since INT is reduced by the ETC prior to cytochrome *c* oxidase^42^, KCN treatment did not affect INT reduction by IMVs energized with either single or combined substrates (Fig. 6). As expected, TRZ activity depended on the specific substrate(s) used to energize the IMVs: none, potent or intermediate inhibition was observed in the presence of succinate (Fig. 6a), NADH (Fig. 6b), or both substrates (Fig. 6c), respectively. In stark contrast, 78.125–125 µM 2B8 potently inhibited the ETC activity when initiated by succinate (Fig. 6a), NADH (Fig. 6b) or both (Fig. 6c). In fact, out of all the tested drugs, 2B8 was the only one to potently inhibit ETC activity initiated by succinate. Again, CCCP had a tendency to increase ETC activity (Fig. 6, but was not significant), whereas 125 µM RA13 only affected INT reduction in the presence of both substrates (Fig. 6c). Supplementary Table S2 summarizes the effects of different compounds on mycobacterial bioenergetics. In sum, high concentrations of 2B8 inhibited the ETC regardless of the substrate(s) energizing *M*. *smegmatis* IMVs, hence its effect is not due to a selective inhibition of NADH dehydrogenases. Moreover, 2B8 compromises mycobacterial bioenergetics differently than a classical uncoupler such as CCCP.

### β-lactam potentiation by drugs targeting mycobacterial bioenergetics

Collectively, these studies confirmed the initial hypothesis that 2B8 targeted mycobacterial bioenergetics. Therefore, it was important to determine whether other compounds targeting mycobacterial bioenergetics, could recapitulate our initial observation that 2B8 potentiates β-lactams^29^. As shown in Table 2, β-lactam potentiation was stronger for 2B8, followed by TRZ and CCCP. In contrast, the effect was lower for BDQ and negligible for RA13.

**Table 2:** Fold β-lactam potentiation with Bioenergetics-Targeting Compounds^a^.

Fold potentiation					
β-lactam	2B8	RA13	CCCP	BDQ	TRZ
Carbenicillin	64x	1.5x	9.6x	4x	16x
Meropenem	64x	2x	16x	4x	32x

^a^Bioenergetics-Targeting Compounds were used at 50% of the MIC reported in Table 1.

Experiments were repeated three times and performed in triplicate.

## Discussion

PMF and bioenergetic homeostasis provide electrochemical gradients and high-energy bonds required for bacterial physiology and survival^10^. Based on our previous observation that 2B8, a cationic amphiphile, affected energy-dependent processes such as protein secretion and lipid export (but not lipid biosynthesis)^29^, it was hypothesized this anti-biofilm compound potentiated β-lactams by collapsing components of the mycobacterial PMF. Using a series of mechanistic studies with intact mycobacteria or IMVs, it is shown herein that 2B8 collapses both components of the PMF and inhibits the ETC, collectively disturbing mycobacterial bioenergetics. Indeed, 2B8 affected multiple bioenergetics parameters including redox homeostasis, both components of the PMF (Δψ and ΔpH), oxygen consumption, ATP generation, ETC activity, NADH/NAD^+^ ratio and NADH oxidation (Supplementary Table S2). These results plausibly explain the diverse, yet related effects induced by 2B8 such as biofilm disruption^28,43^, reversal of antibiotic tolerance^28^, and antibiotic potentiation^29^. Furthermore, it provides a tool to develop compounds with improved adjunct activity that could shorten antimicrobial therapy in TB and other infectious diseases.

2B8 differentially affected mycobacterial bioenergetics parameters in a concentration-dependent manner (Supplementary Table S3). Specifically, whereas uncoupling activity was observed below 62.5 µM, higher concentrations predominantly inhibited the ETC. Similar to other weak cationic amphiphiles^32^, the uncoupler activity is probably due to the ionizable group in 2B8’s aminoimidazole moiety (pKa~8.5). Even though this headgroup is also present in RA13 and other inactive 2-AI compounds^28^, their overall potency is additionally dictated by the structure of different alkyl chains, covalently attached to the linker unit^43^. For instance, in the inactive RA13 it consists of a single chain, thirteen carbons in length (Supplementary Fig. S1). Meanwhile, a shorter and branched alkyl chain is present in 2B8 (Supplementary Fig. S1). These modifications do not significantly impact their hydrophobicity (logD value at pH 7.4 for 2B8 and RA13 is 3.6 and 3.65, respectively), previously shown to correlate with uncoupling activity in cationic amphiphiles^32^. Instead, the shorter and/or branched alkyl chains in 2B8 could confer an enhanced ability to either traverse the mycobacterial cell envelope, partition in and/or flip-flop across the cell membrane to collapse the mycobacterial PMF and interfere with the ETC.

Compelling evidence indicate 2B8’s mechanism of action is not limited to uncoupling activity. In fact, 2B8’s MIC against *M*. *tuberculosis* (Table 1), correlates with the higher dose required to inhibit mycobacterial ETC, rather than the lower dose inducing uncoupling effects. Taking into consideration the importance of PMF on bacterial physiology, this finding was rather surprising and needs further investigation. Nevertheless, 2B8’s effect on mycobacterial bioenergetics (PMF collapse and inhibition of the ETC), conforms to the strategy “uncoupler + additional target” proposed for cationic amphiphiles^32,44^, and currently being unveiled in both traditional and novel drugs. This is the case for pyrazinamide^45,46^, a first-line antibiotic currently used to treat TB. Moreover, TRZ was shown to collapse *Staphylococcus aureus* PMF^47^, in addition to the well-characterized inhibition of NDH-2. Herein, a similar result was obtained when evaluating TRZ effects on mycobacterial bioenergetics. Importantly, significant mycobacterial uncoupling activity was recently described for the novel compounds BDQ^35,36^ and SQ109^48,49^, previously shown to target the mycobacterial ATP synthase^12^ and the mycolate transporter MmpL3^50,51^, respectively. Inhibition of menaquinone biosynthesis was actually identified as a third target for SQ109^49^. Hitting numerous targets has several repercussions in drug research and development. It broadens the antimicrobial spectrum, as reported for SQ109^52^, nitazoxanide^53^ and 2-AI compounds^54^. Furthermore, it decreases the likelihood of selecting for resistant mutants. Indeed, in the order of >10^12^ bacteria were required to isolate mutants resistant to nitazoxanide^53^, whereas mycobacterial mutants to SQ109 have not been isolated^50^.

**Figure 7:**
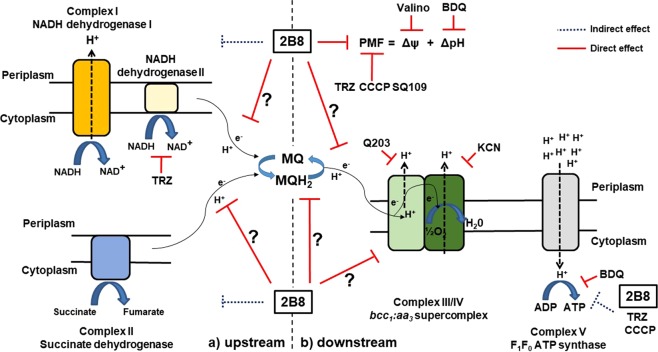
Diagram of 2B8’s effect on mycobacterial bioenergetics. Known bioenergetics-affecting drugs are noted as well. Solid and dotted lines indicate direct and indirect targets, respectively. The question marks indicate additional sites potentially inhibited by 2B8, located at either side of menaquinone reduction (black dotted line). (**a**) Upstream of menaquinone reduction. 2B8 inhibits the function of NADH and succinate dehydrogenases: catalyze menaquinone reduction as NADH and succinate are oxidized, respectively. CFZ restores NADH oxidation by NDH-2 but not by the other types of NADH dehydrogenases or succinate dehydrogenase. (**b**) Downstream of menaquinone reduction. 2B8 blocks the ETC downstream of NADH and succinate dehydrogenases (after the pathways converge), but upstream of complex IV. Specifically, 2B8 interferes with menaquinol oxidation and/or diffusion, or with complex III.

How 2B8 blocks mycobacterial ETC remains to be fully defined. Despite several attempts, resistant mutants have not been successfully obtained. However, this might require evaluating higher mycobacterial numbers as discussed above. The probability of isolating resistant mutants is also determined by the nature of the target(s), being less feasible if membrane function rather than enzymatic activity is compromised^10^. As reported for other cationic amphiphiles^32^, the hydrophobic moiety in 2B8 could mediate drug-membrane interactions affecting membrane packaging and/or lipid diffusion. These more subtle and acute effects on membrane physiology could affect the ETC and precede any evidence of the catastrophic mycobacterial cell membrane disruption observed only after prolonged incubation (24 h) with 2B8 ≥ 125 µM^29^. That 2B8 but not TRZ-mediated inhibition of NADH oxidation was partially restored by CFZ, ruled out NDH-2 as the target for 2B8. Furthermore, the fact that 2B8 inhibited INT reduction when the mycobacterial ETC was energized with either succinate, NADH (despite not directly inhibiting NDH-2), or both substrates suggests at least two distinct scenarios (Fig. 7): (a) Upstream of menaquinone reduction. 2B8 simultaneously interferes with succinate and NADH oxidation by blocking menaquinone reduction at complex II and the three types of mycobacterial NADH dehydrogenases, respectively. Adding CFZ only restores NADH oxidation catalyzed by NDH-2 and the degree of restoration is proportional to CFZ’s ability to compete with 2B8 for NDH-2; (b) Downstream of menaquinone reduction. 2B8 targets the ETC downstream of succinate and NADH dehydrogenases, after these pathways converge. However, since KCN and 2B8 had different effects on INT reduction, it is unlikely 2B8 targets the cytochrome *aa*_*3*_ subunit present in complex IV. Thus, potential sites blocked by 2B8 would be narrowed to: menaquinol oxidation and/or diffusion, or complex III (cytochrome *c* reductase). Ongoing experiments are being performed to elucidate this.

Experiments are currently underway to determine how mycobacteria withstand 2B8’s collapse of the PMF. Transcriptomics analysis indicated that in response to 2B8^29^, *M*. *tuberculosis* upregulated genes previously shown to be involved in drug efflux such as *mmpL5*^55^, *rv1218c* and *rv1258c*^56^, or detoxification such as *rv3161c*^57^. However, taking into consideration the fact that efflux pumps require PMF^58^, it is unlikely this constitutes a major mechanism counteracting 2B8’s effects. Instead, mycobacteria could inactivate 2B8 by modifying the ionizable headgroup and/or the alkyl chains, jointly required for its activity. This is currently being evaluated to determine if 2B8’s stability and/or efficacy is amenable to improvement via medicinal chemistry. Transcriptomics analysis also provided evidence that 2B8 induced remodeling of mycobacterial metabolism^29^. Mycobacterial carbon and nitrogen metabolism was altered by 2B8 treatment, as suggested by the upregulation of *icl1*, *prpC* and *prpD* (involved in the methylcitrate cycle^59^), and downregulation of *glnA1* and *pknG* (involved in the biosynthesis and regulation of glutamine and glutamate^60,61^), respectively. Interestingly, it was previously shown that metabolic remodeling endowed *M*. *tuberculosis* the ability to survive for four days in the presence of BDQ at 300 × MIC^39^, a concentration determined herein to uncouple mycobacteria, in agreement with the initial results from Cook^35^ and later confirmed by Steyn^36^. The metabolic adaptations induced by BDQ and 2B8 are very different and it remains to be determined if they play a role to counteract 2B8. However, this highlights the fact that mycobacteria are able to temporarily persist in the presence of uncoupling concentrations of an excellent drug such as BDQ (or for that matter, 2B8). Moreover, it emphasizes that uncoupling might not be sufficient against TB and when considering the strategy “uncoupler + additional target”, the second component could be just as important or even more.

Lastly, the ability to potentiate β-lactam antibiotics against *M*. *tuberculosis* was compared between several compounds targeting mycobacterial bioenergetics at different sites (Table 2). Not surprisingly, carbenicillin and meropenem were particularly potentiated by TRZ, a compound affecting both mycobacterial bioenergetics and cell envelope permeability^29,62^, similar to 2B8. Meanwhile, β-lactam potentiation by CCCP was intermediate. This could be envisioned as the maximum β-lactam potentiation achieved by a compound principally acting as an uncoupler. Conversely, the weak β-lactam potentiation observed with BDQ at 0.5 × MIC could be attributed to the fact that BDQ does not have uncoupling activity in mycobacteria at this lower concentration^36^. In sum, at the tested drug concentrations, the magnitude of β-lactam potentiation (2B8 > TRZ > CCCP) could be collectively determined by: a) effects on mycobacterial bioenergetics (CCCP > TRZ~2B8), plus b) enhanced mycobacterial cell envelope permeability (only induced by 2B8 and TRZ^29,62^), conducive to increased β-lactam penetration in 2B8-treated *M*. *tuberculosis*^29^. Ongoing experiments are being performed to determine if this concerted effect on mycobacterial bioenergetics and cell envelope permeability, is the mechanism behind 2B8’s anti-biofilm properties.

It should be noted that whole-cell experiments were performed mainly in *M*. *tuberculosis*, whereas assays with IMVs were exclusively performed using *M*. *smegmatis*-derived membranes. Even though using an avirulent mycobacteria could be a limitation to our study, for the following reasons we consider *M*. *smegmatis* a valid source of mycobacterial membranes not significantly impacting the conclusions: a) whole-cell assays using *M*. *smegmatis* or *M*. *tuberculosis* gave similar results. This was the case for the membrane potential assay presented herein using *M*. *smegmatis* and DisC_3_(5) (Fig. 1), additionally evaluated by flow cytometry and DiOC_2_(3) staining in *M*. *tuberculosis* (not shown). The former is presented because it has the benefit of being a real-time assay; b) the overall potency of the tested compounds did not differ significantly between assays using *M*. *tuberculosis* whole-cells and *M*. *smegmatis* IMVs; c) there was a good correlation between both types of assays. For instance, the *in vivo M*. *tuberculosis* NADH/NAD^+^ ratio was increased by TRZ, 2B8 and BDQ, but tended to decrease in the presence of CCCP (Fig. 4). These results paralleled the effects these compounds had on NADH oxidation (Fig. 5) and electron transport (Fig. 6) in *M*. *smegmatis* IMVs; d) our results with *M*. *smegmatis* IMVs evaluating restoration of NADH oxidation by CFZ (Fig. 5) and BDQ’s effect on NADH oxidation (Fig. 5), are similar to those reported for *M*. *tuberculosis* IMVs^20,36^; and e) due to the inside out membrane topology in IMVs, the mycobacterial inner membrane would be external and readily accessible to the compounds. Thus, the difference in *M*. *smegmatis* and *M*. *tuberculosis* cell envelope composition^3–5^, known to impact drug diffusion and probably contributing to 2B8’s higher MIC against *M*. *tuberculosis*^29^, is bypassed in IMVs. Indeed, this could explain why 2B8’s inhibitory effect on the ETC of *M*. *smegmatis* IMVs was observed at concentrations ≥78 μM (Fig. 6), whereas higher concentrations (≥93 μM) were required to decrease OCR (Fig. 2). It is however, possible that subtle differences reported in the ETC of these related organisms^18^, could have some repercussions on the experimental outcome.

In conclusion, it was determined that 2B8 collapses both components of the mycobacterial PMF and interferes with the ETC. Further investigation into the mechanism of action of 2B8 could lead to the elucidation of novel drug targets in *M*. *tuberculosis* and increase our knowledge in the field of mycobacterial bioenergetics.

## Methods

All materials were purchased from Sigma-Aldrich (St. Louis, MO, USA), unless indicated.

### Bacterial strains, media, and culture conditions

*M*. *smegmatis* mc^2^155 and the BSL2 strain *M*. *tuberculosis* H37Rv mc^2^ 6206 were grown as before^29^. *M*. *tuberculosis* H37Rv mc^2^ 6206 was a kind gift from Dr. William R. Jacobs Jr. at Albert Einstein College of Medicine.

### Kinetic measurement of alamarBlue^®^ reduction

*M*. *smegmatis* and *M*. *tuberculosis* cultures were adjusted to a specific OD_600_ (0.4 for *M*. *smegmatis* and 1.0 for *M*. *tuberculosis*), and 100 µL of each culture was distributed per well to a clear-bottom, black-wall, 96-well plate (Corning, Corning, NY USA). Cultures were treated with DMSO control, 7.825–250 µM 2B8 and RA13 (provided by Dr. Christian Melander), 15 µM carbonyl cyanide m-chlorophenyl hydrazine (CCCP), 80 µM TRZ or 18 µM BDQ (BOC Sciences, Shirley, NY, USA). Immediately after treatment, 10 µL of alamarBlue^®^ reagent (Life Technologies, Carlsbad, CA, USA) was added to each well. Using a Biotek Synergy 2 multi-mode plate reader (Biotek, Winooski, VT, USA) set at 37 °C, fluorescence at 530_ex_/590_em_ was measured over time, as indicated in the figures.

### Determination of Δψ collapse by DisC_3_(5)

The collapse of *M*. *smegmatis* Δψ was evaluated with the fluorescent, membrane potential sensitive probe 3,3′-dipropylthiodicarbocyanine (DisC_3_(5), Life Technologies), as previously described with minor modifications^48,49^. *M*. *smegmatis* culture was adjusted to an OD_600_ of 0.3. Prior to the assay, dextrose and nigericin were added to a final concentration of 10 mM and 1 µM, respectively. One hundred µL of culture was distributed per well in a clear-bottom, black-wall 96-well plate (Corning, Corning, NY, USA), followed by addition of DisC_3_ (5) at a final concentration of 5 μM. DisC_3_(5) quenching due to bacterial uptake was monitored by fluorescence at 600_ex_/680_em_, 30 °C in a Biotek Synergy HT multi-mode plate reader. Once fluorescence was quenched, bacteria were treated with DMSO, different concentrations of 2-AI compounds (2B8 and RA13: 31.25–125 µM), 15 µM CCCP, 18 µM BDQ, or 80 µM TRZ. Plates were continuously monitored to test for fluorescence reversal when DisC_3_(5) is released, an indicator of bacterial membrane potential (Δψ) depolarization.

### Real-time measurement of oxygen consumption rate by high-resolution respirometry

Mycobacterial oxygen consumption in the presence or absence of 2-AI compounds or other drugs was monitored in real-time using Oroboros Oxygraph-2k (http://www.oroboros.at, Oroboros Instruments, Innsbruck, Austria). The chambers of Oroboros Oxygraph-2k were filled with 2.5 mL of 7H9 supplemented with 0.2% dextrose and glycerol. Basal oxygen level (nmol/mL) and oxygen consumption rate (OCR) (pmol/s × mL) without bacteria was measured and calibrated using Datlab4 software (Oroboros Instruments). Thereafter, mid-log phase *M*. *smegmatis* or *M*. *tuberculosis* cultures were adjusted to an OD_600_ of 0.5 in the same media used to fill the chambers. Finally, 100 or 400 µL of *M*. *smegmatis* or *M*. *tuberculosis* respectively, was injected into the Oroboros Oxygraph-2k chambers with a Hamilton Microliter Syringe (Hamilton Company, Reno, NV, USA) and the OCR level of non-treated bacteria was recorded. Continuous OCR monitoring was performed upon addition of increasing concentrations of 2-AI compounds, CCCP, BDQ or TRZ, as indicated in each experiment. Fold change in OCR levels were calculated by dividing OCR in the presence of compounds, by basal OCR (before adding compounds). This assay was performed in media with or without bovine serum albumin (BSA). When performing experiments with albumin, media was supplemented with 0.5% fatty acid-free BSA (Sigma-Aldrich).

### Quantification of intracellular ATP

*M*. *tuberculosis* was cultured to an OD_600_ of 0.5 and treated with DMSO, three concentrations of 2B8 and RA13 (31.25, 62.5, and 125 µM), 15 µM CCCP, 18 µM BDQ, or 80 µM TRZ. Treated cultures were incubated at 37 °C, for 2 or 24 h under agitation. Thereafter, cultures were centrifuged at 1,700 × g, washed once with sterile PBS and reconstituted in 500 µL of PBS. An aliquot of washed culture was plated on 7H11 agar (BD, USA) for colony forming units (CFU) enumeration after 3–4 weeks of culture at 37 °C. The remaining culture was transferred to screw-cap 2 mL microtubes (Fisher Scientific, Waltham, MA, USA) containing 100 µm Zirconia beads (Biospec, Bartlesville, OK, USA). Bacteria were lysed by bead-beating six times for 30 sec at maximum speed and cooling in ice for one min in between. Samples were briefly centrifuged to remove precipitates and 100 µL of supernatant was transferred to a clear-bottom, white-wall 96-well plate (Corning, USA). ATP concentration was quantified using BacTiter-Glo^TM^ Microbial Cell Viability Assay (Promega, Madison, WI, USA) as previously described with some modifications^13^. Briefly, an equal volume of BacTiter-Glo^TM^ reagent was added to the samples. After shaking the plate at RT for 5 min, luminescence at 550 nm was recorded using a Biotek Synergy 2 multi-mode plate reader. Relative luminescence units (RLUs) was divided by CFUs and the data is presented as RLUs/CFUs.

### Determination of NADH/NAD^+^ ratio

The NADH/NAD^+^ ratio was determined as previously described with minor modifications^63^. *M*. *tuberculosis* H37Rv mc^2^ 6206 was cultured to an OD_600_ of 0.5. One ml cultures were treated with the following compounds: three different concentrations of 2B8 or RA13 (31.25, 62.5, 125 µM), 15 µM CCCP, 18 µM BDQ, or 80 µM TRZ. After treatment for 2 or 24 h at 37 °C, cultures were split into two-500 µL aliquots for separate extraction of NADH and NAD^+^. Bacteria were pelleted by centrifugation at 1,700 × g and the supernatant was removed. Three hundred µL of 0.2 M HCl (for NAD^+^ extraction) or 0.2 M NaOH (for NADH extraction) was added and placed in a 55 °C water bath for 10 min, followed by immediate cooling in ice. After cooling, sample pH was neutralized with 300 µL 0.1 M NaOH (for NAD^+^ extraction) or 0.1 M HCl (for NADH extraction). Precipitates were removed via centrifugation and 50 µL of supernatant was transferred to a clear-bottom, 96-well plate (Corning, USA). NADH or NAD^+^ was quantified using the NADH/ NAD^+^ quantification kit from Sigma-Aldrich per the manufacturer’s instructions. Briefly, 100 µL of NAD^+^ cycling enzyme mix was added to the samples and incubated for 5 min at RT, under constant shaking to convert NAD^+^ to NADH. Thereafter, 10 µL of NADH developer was added to each well and incubated at RT until color developed (1–2 h). End point absorbance at 450 nm was measured using a Biotek Synergy multi-mode plate reader (Biotek, USA) and the NADH/NAD^+^ ratio was calculated.

### β-lactam potentiation assay

β-lactam potentiation assay was performed as previously described^29^. Briefly, the minimum inhibitory concentration (MIC) of nigericin, valinomycin, CCCP, BDQ and TRZ against *M*. *tuberculosis* was determined. Thereafter, the MIC of carbenicillin or meropenem against *M*. *tuberculosis* was determined in the presence or absence of nigericin, valinomycin, CCCP, BDQ and TRZ at a concentration equivalent to 50% of their respective MIC. β-lactam potentiation was calculated by dividing the MIC of each β-lactam by the MIC of each β-lactam plus drug.

### Generation of IMVs

Preparation of IMVs from *M*. *smegmatis* cells was done as previously described with minor modifications^14^. Briefly, 5–10 g wet weight of *M*. *smegmatis* were resuspended at a 1:2 ratio (wt/vol) in breaking buffer (50 mM MOPS pH 7.5, 2 mM MgCl_2_) and protease inhibitor cocktail (Roche, Basel, Switzerland). The suspension was stirred for 1 h at room temperature in the presence of 1.2 mg/mL lysozyme. The MgCl_2_ concentration was adjusted to 15 mM and 0.2 mg/mL DNase I was added. Bacteria were lysed by passing six times through a pre-chilled French Press at 20,000 psi (Thermo Electron, Waltham, MA, USA). The lysate was centrifuged at 3,000 × g for 30 min to pellet unbroken cells and the supernatant was further centrifuged at 27,000 × g for 30 min to pellet cell wall. The supernatant was harvested and centrifuged for 1 h at 100,000 × g using an ultracentrifuge (Beckman Coulter, Brea, CA, USA), to pellet IMVs. After removing the supernatant, pelleted IMVs were resuspended in breaking buffer and the protein concentration was measured using Pierce BCA protein assay (Thermo Scientific, Waltham, MA, USA). Glycerol was added to a final concentration of 10% and aliquots of IMVs stored at −80 °C until further use.

### Determination of ΔpH collapse with IMVs

Collapse of ΔpH was determined in *M*. *smegmatis* IMVs as previously described with minor modifications^35^. The pH-sensitive, fluorescent dye 9-amino-6-chloro-2-methoxyacridine (ACMA, Life Technologies) was used instead of acridine orange. IMVs were diluted to 0.1125 mg/mL in 10 mM HEPES (pH 7.5), 100 mM KCl, 5 mM MgCl_2_ and added to a black-wall, 96-well plate (Corning). IMVs were pre-incubated with 2 µM ACMA at 37 °C for 30 minutes and the baseline 410_ex_/460_em_ fluorescence was measured using a Biotek Synergy HT multi-mode plate reader. IMVs were energized with 5 mM NADH and incubated until ACMA fluorescence was quenched due to generation of ΔpH. Thereafter, IMVs were treated with DMSO, different concentrations of 2-AI compounds (2B8 and RA13: 31.25–125 µM), 15 µM CCCP, 18 µM BDQ, 80 µM TRZ, or 10 µM nigericin, and monitored for 4 minutes to test for fluorescence reversal if ΔpH collapsed.

### ETC activity assay with IMVs

The ETC activity was measured as previously described with minor modifications^64^. Briefly, *M*. *smegmatis* IMVs were resuspended at 0.1125 mg/mL in 10 mM HEPES (pH 7.5) buffer. Twenty five µL of suspended IMVs were distributed in a clear-bottom, transparent 96-well plate (Corning), containing an equal volume of 4 mM 2-(*p*-iodophenyl)-3-(*p*-nitrophenyl)-5-phenyl tetrazolium chloride (INT). Drug treatments were made by adding DMSO, four concentrations of 2B8 (62.5 µM to 125 µM), 125 µM RA13, 15 µM CCCP, 18 µM BDQ, 80 µM TRZ, and 5 mM potassium cyanide (KCN). ETC activity was initiated by adding 75 µL of 0.2% Triton X-100 in PBS supplemented with either 1 mM NADH, 130 mM sodium succinate or both. Absorbance at 490 nm was immediately monitored at 37 °C for 10 minutes using a SpectraMax M series multi-mode plate reader (Molecular Devices, Sunnyvale, CA, USA).

### Determination of NADH oxidation by IMVs

NADH oxidation was measured using fluorescence emission at 460 nm when excited at 340 nm as previously described^41^. *M*. *smegmatis* IMVs were resuspended at 0.1125 mg/mL in 10 mM HEPES (pH 7.5), 100 mM KCl, 5 mM MgCl2 (Sigma-Aldrich, USA) and 1 mM NADH. One hundred µL of suspended IMVs were distributed in a clear-bottom, black-wall 96-well plate (Corning), containing DMSO, four concentrations of 2B8 (62.5 µM to 125 µM), 125 µM RA13, 15 µM CCCP, 18 µM BDQ, 80 µM TRZ, or 5 mM KCN. Thereafter, fluorescence (340_ex_/460_em_) was monitored using a Biotek Synergy HT multi-mode plate reader at 37 °C for 9 minutes.

### Restoring NADH oxidation with CFZ

NADH oxidation assay was performed as described above. *M*. *smegmatis* IMVs were incubated with 1 mM NADH in the presence of 125 µM 2B8, 80 µM TRZ or 5 mM KCN and NADH oxidation was monitored for 2 min. Thereafter, to determine if CFZ could restore NADH oxidation by acting as an alternative electron acceptor for NDH-2^20^, 42 µM CFZ or DMSO was added and NADH oxidation was monitored for an additional 10 minutes. CFZ was added a second time and NADH oxidation monitored as above.

### Statistical analysis

Statistical analyses were carried out using Student t-test or one way ANOVA with Tukey’s post hoc test using GraphPad Prism 5 (GraphPad Software, La Jolla, CA, USA). P-values less than 0.05 were considered significant.

## Supplementary information

Supplementary Information

## Data Availability

The datasets generated during and/or analysed during the current study are available from the corresponding author on reasonable request.
