# Supplementary material for: 2-aminoimidazoles collapse mycobacterial proton motive force and block the electron transport chain
Source: Sci Rep. 2019 Feb 6;9:1513. doi: 10.1038/s41598-018-38064-7 (PMC6365497; doi:10.1038/s41598-018-38064-7)
Supplement: Supplementary file 1 — Supplementary Information [file 41598_2018_38064_MOESM1_ESM.pdf]

2-aminoimidazoles collapse mycobacterial proton motive force and block the electron transport chain

Albert Byungyun Jeon[1,a], David F. Ackart[1], Wei Li[1], Mary Jackson[1], Roberta J. Melander[2,b], Christian Melander[2,b], Robert B. Abramovitch[3], Adam J. Chicco[4], Randall J. Basaraba[1,*], Andrés Obregón-Henao[1,*]

Figure S1.

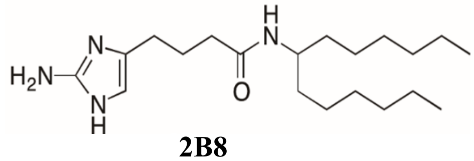

2B8

Active

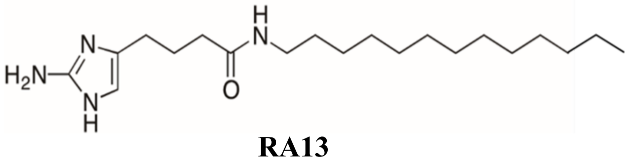

RA13

Inactive

Figure S1. Structures of 2-AI compounds.

Figure S2.

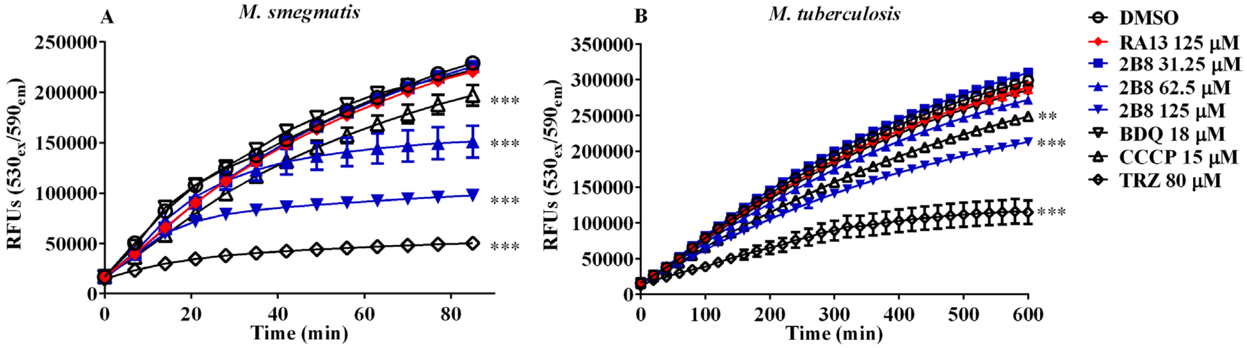

Figure S2. 2B8 inhibits alamarBlue® reduction by mycobacteria. *M. smegmatis* and *M. tuberculosis* were treated with 2-AI compounds or drugs targeting mycobacterial bioenergetics, while being monitored for alamarBlue® reduction immediately following treatment. A) After 80 min, 62.5 and 125 µM 2B8, 15 µM CCCP, and 80 µM TRZ inhibited alamarblue® reduction by *M. smegmatis*. B) AlamarBlue® reduction by *M. tuberculosis* was inhibited with 15 µM CCCP, 80 µM TRZ and 125 µM 2B8 (but not 62.5 µM). For both *M. smegmatis* and *M. tuberculosis*, incubation with 125 µM RA13 and 18 µM BDQ did not have an effect. Experiments were repeated three separate times and representative data are shown. * $p < 0.05$, ** $p < 0.01$, *** $p < 0.001$ by ANOVA.

Table S1. MICs of 2-AI compounds against *M. smegmatis* and *M. tuberculosis* in the presence or absence of albumin.

	M. tuberculosis H37Rv mc^2 6206		*M. smegmatis*	
	+0.5% BSA	No BSA	+0.5% BSA	No BSA
2B8	250-500	250	125-250	62.5
RA13	> 1000	> 1000	> 1000	> 1000

All MIC values are μM.

Table S2: Summary of bioenergetics effects induced by tested compounds.

Read-out	2B8	RA13	CCCP	BDQ	TRZ	KCN
**Mechanism of action**	Uncoupler & ETC block at unknown site	Negligible	Uncoupler & Proton ionophore	ATP synthase Inhibitor & Uncoupler	NDH-2 inhibitor & Uncoupler	ETC block at cytochrome c oxidase
**alamarBlue® reduction**	Strong ↓	No effect	Weak ↓	No effect	Strong ↓	N/T
**Δψ collapse**	Strong	No effect	Strong	No effect	Intermediate	N/T
**ΔpH collapse**	Strong	Weak	Intermediate	Intermediate	Strong	N/T
**O2 consumption rate**	↑ then ↓	Weak ↑	↑ then ↓	↑	↑	N/T
**[ATP]i**	Strong ↓	No effect	Intermediate ↓	Strong ↓	Strong ↓	N/T
**ETC activity**	Strong ↓	Weak ↓	Weak ↑	No effect	Strong ↓	No effect
**NADH/NAD+ ratio**	Strong ↑	No effect	No effect	Intermediate ↑	Strong ↑	N/T
**NADH oxidation**	Strong ↓	No effect	Weak ↑	Intermediate ↓	Strong ↓	Strong ↓
**Rescue by CFZ**	Partial	N/T	No effect	N/T	No	Yes
**ß-lactam potentiation**	Strong ↑	Marginal ↑	Intermediate ↑	Weak ↑	Intermediate ↑	N/T

↓= Inhibition
↑= Induction
N/T= Not tested

Table S3. Comparison of the effects induced by a low or high concentration of 2B8.

	Low (≤ 62.5 µM)	High (> 62.5 µM)
**Proposed action**	Uncoupler	ETC blocker
**Acute (≤ 2 h)**		
**Δψ collapse**	Strong	Strong
**ΔpH collapse**	Low-Strong	Strong
**O2 consumption rate**	↑	↓ to basal
**ETC activity**	No effect	↓
**NADH oxidation**	No effect	↓
**[ATP]i**	No effect	No effect
**NADH/NAD+ ratio**	No effect-↑	↑
**Delayed (> 2 h)**		
**alamarBlue® reduction**	No effect	↓
**[ATP]i**	No effect-↓	↓
**NADH/NAD+ ratio**	No effect-↑	↑

↓= Inhibition
↑= Induction
